# Periocular Reconstruction in Facial Trauma: Surgical Approaches and Multidisciplinary Perspectives

**DOI:** 10.7759/cureus.91344

**Published:** 2025-08-31

**Authors:** Fernando Fernández Varela Gómez, Katherine López Soto, Reinhard A Ortiz Beitz, Víctor Hugo Garzón Ortega, Alec Anceno, Kevin J. Fuentes Calvo, Kenzo Alejandro Fukumoto Inukai, Samantha Palomino Rangel, Fidel Iván Flores Pérez, María Paola Orozco Náder, Mauricio G. Padilla Sierra

**Affiliations:** 1 Department of Surgery, Faculty of Medicine, Universidad Nacional Autónoma de México, Mexico City, MEX; 2 Internal Medicine, Hospital Central Sur de Alta Especialidad, Mexico City, MEX; 3 Department of Ophthalmology, Faculty of Medicine, Universidad Nacional Autónoma de México, Mexico City, MEX; 4 Department of Surgery, Christus Muguerza Hospital Alta Especialidad, Monterrey, MEX; 5 Department of Urology, University of Southern California, Los Angeles, USA; 6 Department of Surgery, Hospital Médica Sur, Mexico City, MEX; 7 Department of Ophthalmology, Universidad Panamericana, Mexico City, MEX; 8 Plastic and Reconstructive Surgery, Hospital Ángeles Pedregal, Mexico City, MEX

**Keywords:** facial trauma, multidisciplinary collaboration, orbital fractures, periocular reconstruction, reconstructive techniques

## Abstract

Periocular reconstruction after facial trauma addresses the intricate anatomy and essential functions of the eye region. This summary explores the epidemiology, classification, preoperative planning, timing, and reconstructive strategies for periocular injuries. Classification by the type of injury and the affected structure informs surgical approaches, supported by detailed imaging and clinical assessments. Timing of reconstruction ranges from immediate action in critical cases to delayed procedures when stability permits. Techniques like local flaps, grafts, microvascular transfers, and innovations such as 3D-printed implants are customized to each defect. Key priorities include restoring the eyelid margin, preserving the lacrimal system, and adapting to age-specific needs, such as in children or older adults. Multidisciplinary teamwork enhances perioperative care, focusing on infection control and inflammation management. While advances improve outcomes, challenges remain in standardizing results and determining optimal intervention timing, pointing to areas for future exploration in periocular trauma care.

## Introduction and background

Facial trauma affecting the periocular region-encompassing the eyelids, orbit, and surrounding structures-presents unique challenges in reconstructive surgery due to its complex anatomy and critical functional roles [1]. Effective reconstruction requires a deep understanding of the epidemiology, common causes, and anatomical and functional factors guiding surgical intervention. This narrative review aims to synthesize epidemiological patterns, etiological factors, injury types, and reconstructive considerations in periocular trauma, providing a focused resource for clinicians and trainees.

Epidemiology and etiologies of periocular trauma 

Periocular trauma is a significant global health issue, with an estimated age-standardized incidence rate of approximately 98 per 100,000 population worldwide in 2017 and predominance of male patients (male-to-female ratio ~2:1, with rates of male patients at 130 per 100,000 versus 66 per 100,000 for female patients), linked to their greater involvement in high-risk activities [2]. In the United States, the age-standardized incidence rate for facial fractures reached 151 per 100,000 in 2021, while in China it was around 91 per 100,000, reflecting regional variations [3]. Young adults, especially men aged 20-34, are the most affected group, primarily due to road traffic accidents and interpersonal violence [3]. Children and adolescents also experience a notable incidence, with road traffic accidents, falls, and sports injuries as leading causes [4]. Geographic differences influence these patterns: road traffic accidents dominate in Africa, falls in Asia, and violence or sports injuries in North America [4]. Motor vehicle accidents often result in contusions and abrasions, with associated eye injuries occurring at rates of 2.7 to 3.4 per 100,000 annually in the US [5], while assaults, particularly with blunt objects, are a major cause of orbital floor fractures [6]. Falls commonly affect the elderly and children, causing lacerations and fractures, with fall-related eye trauma presenting at rates of 31 to 34 per 100,000 in US emergency departments [7]. In children, sports injuries, typically managed conservatively, may require intervention if complications occur [8].

Anatomical and functional considerations 

**Figure 1:**
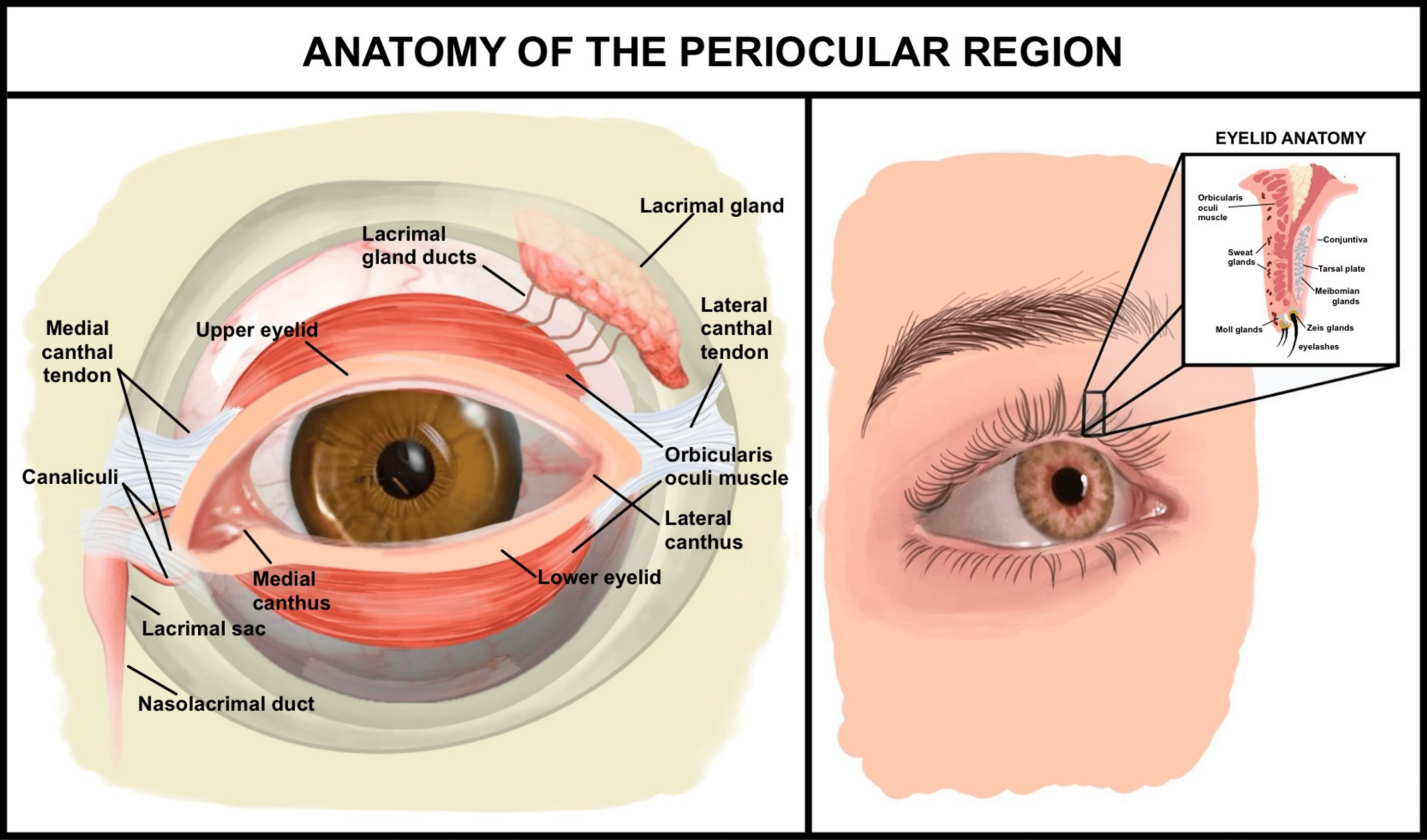
Anatomy of the periocular region The figure illustrates the key anatomical structures of the periocular region, including the upper and lower eyelids, lacrimal apparatus (including the gland, ducts, sac, and nasolacrimal duct), the canthal tendons, and the orbicularis oculi muscle. The inset provides a detailed cross-section of eyelid anatomy, highlighting layers such as the tarsal plate, conjunctiva, meibomian glands, and cilia. Image credit: Created by Katherine López Soto using Procreate (Hobart, Australia).

Successful periocular reconstruction depends on restoring the eyelids’ anterior lamella (skin and orbicularis oculi muscle) and posterior lamella (tarsal plate and conjunctiva), ensuring at least one is vascularized for viability [1,9]. The canthal tendons, which anchor the eyelids to the orbital rim, are essential for maintaining contour and must be precisely reattached if disrupted [9]. The lacrimal drainage system, including the lacrimal sac and nasolacrimal duct, is crucial for tear drainage, and its damage can lead to epiphora, requiring careful repair. [10] These structures collectively support the goals of functional restoration and aesthetic outcomes (Figure 1).

## Review

Classification and mechanisms of periocular trauma

Classification of Periocular Injuries

**Figure 2:**
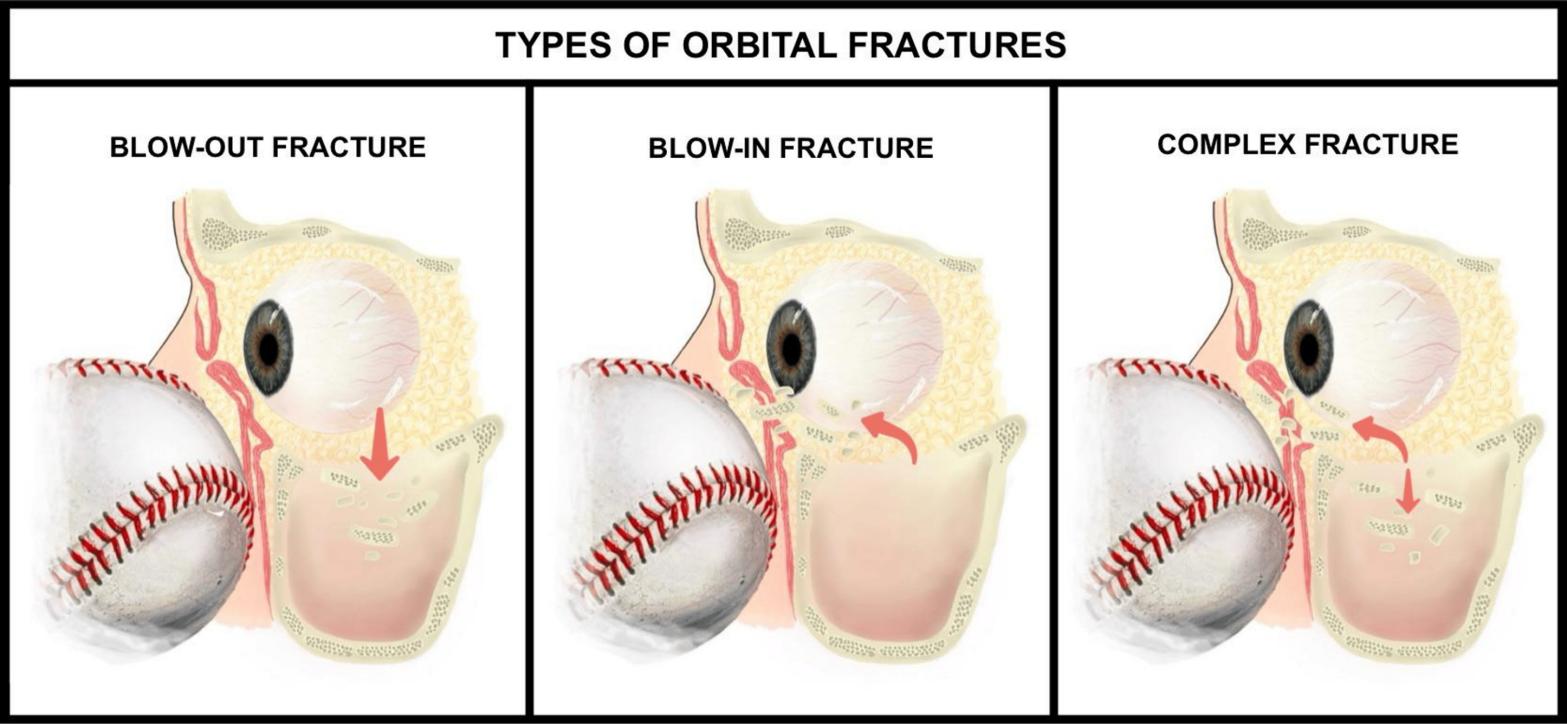
Types of orbital fractures in periocular trauma The figure shows the mechanisms and effects of common orbital fractures from blunt trauma, such as the impact of a baseball. The left panel depicts a blow-out fracture with orbital floor depression and increased volume (risk of enophthalmos); the middle shows a blow-in fracture with inward bone displacement and decreased volume (risk of exophthalmos); and the right panel illustrates a complex comminuted fracture with multiple fragments. Image credit: Created by Katherine López Soto using Procreate (Hobart, Australia).

Periocular injuries are classified based on the mechanism of injury and the structures affected, guiding clinical decision-making. Blunt trauma, caused by non-penetrating forces, often results in contusions, hematomas, or orbital fractures, including isolated blow-out fractures or comminuted disruptions [11,12] (Figure 2).

Penetrating injuries, which breach the skin and deeper tissues, pose a higher risk of severe ocular damage, such as open-globe injuries, despite the less frequent association with orbital fractures [12]. Injuries are further categorized by the structures involved: isolated soft tissue injuries, typically from blunt trauma, present as hematomas or edema without bony involvement [11], while orbital fractures most commonly affect the floor, followed by the medial wall, lateral wall, and the roof [13]. These fractures vary in complexity and impact the orbital volume, requiring detailed imaging, such as CT scans, to assess and guide intervention [14].

Mechanisms of Complex Periocular Damage

**Figure 3:**
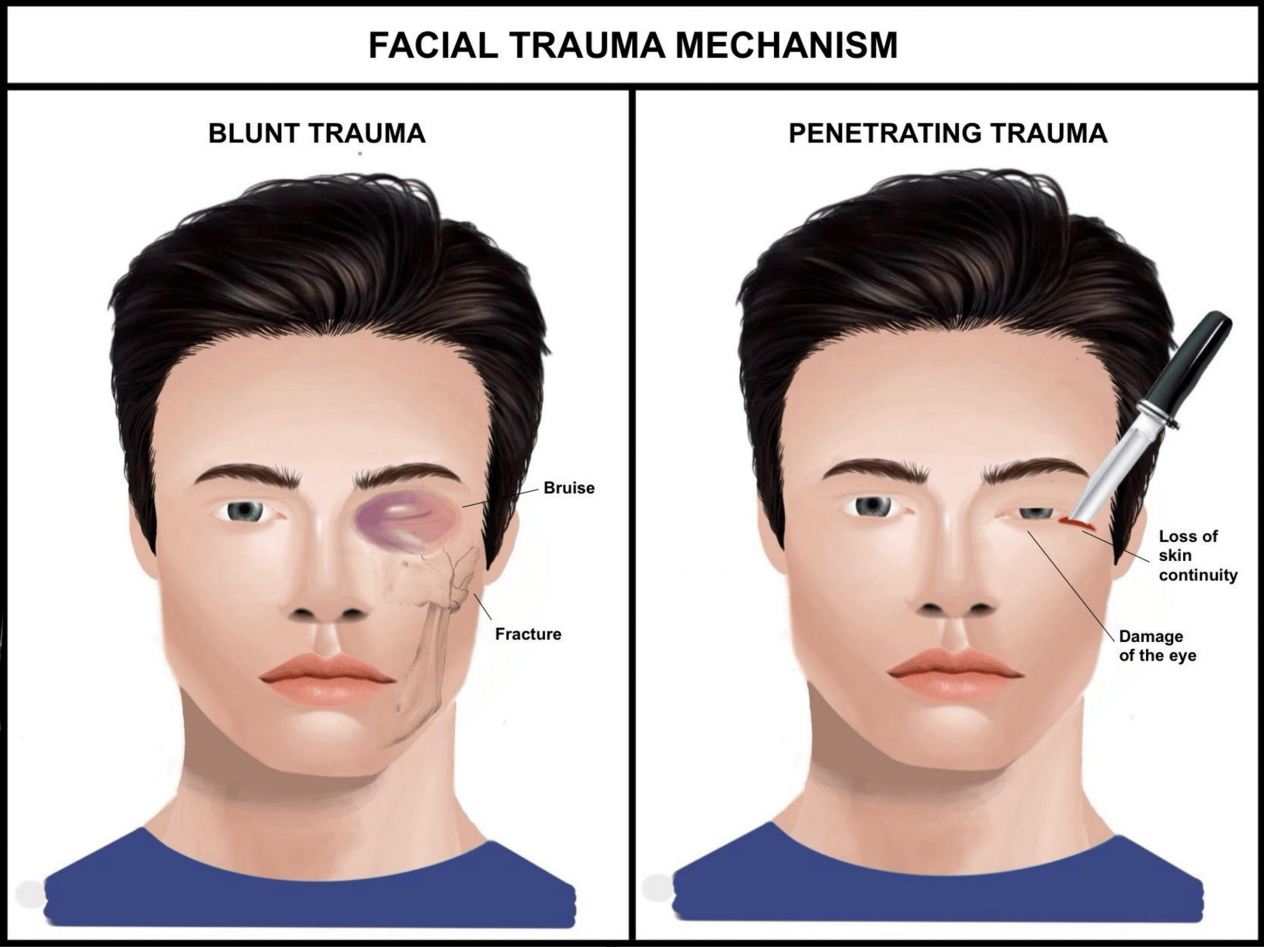
Mechanisms of blunt and penetrating facial trauma This figure contrasts blunt trauma (left panel), resulting in periorbital bruising and underlying fractures, with penetrating trauma (right panel), involving loss of skin continuity and direct damage to the eye and surrounding structures, highlighting the differing injury patterns and risks in periocular trauma. Image credit: Created by Katherine López Soto using Procreate (Hobart, Australia).

Complex periocular injuries, marked by severe functional and aesthetic compromise, often result from high-energy mechanisms. Gunshot wounds, for example, cause orbital fractures and open-globe injuries, carrying a high risk of blindness due to the intense biomechanical forces involved [15]. Sharp trauma from assaults also inflicts significant soft tissue and bony damage, frequently involving ocular injury [16,17] (Figure 3).

These injuries require urgent ophthalmologic evaluation and meticulous assessment to address the interplay of damaged structures and to prevent long-term deficits.

Classification Systems for Surgical Planning

Several classification systems enhance surgical precision by focusing on anatomical subunits and defect characteristics. The aesthetic unit-based approach divides the periorbital region into zones, allowing tailored technique selection, such as local flaps or grafts [18]. A vertical dimension classification for lower eyelid defects categorizes them as pretarsal, preseptal, eyelid-cheek junction, or complex, helping predict postoperative retraction and guiding revisions [19]. These systems refine planning to balance functional restoration and aesthetic outcomes.

Biomechanical Properties Influencing Reconstruction

The biomechanical properties of the periorbital region, particularly in orbital floor fractures, critically influence reconstructive approaches. The periorbita and orbital floor complex exhibit distinct mechanical resistances, suggesting that resorbable materials may suffice for isolated floor fractures to prevent enophthalmos [20]. Measures like orbital fracture area and volume correlate with late enophthalmos, establishing thresholds for surgical intervention [21]. These insights ensure structural and aesthetic integrity in reconstruction. The classification of periocular injuries can be systematically organized based on key criteria, providing a foundation for surgical decision-making (Table 1).

**Table 1:** Classification of periocular injuries The table summarizes the classification of periocular injuries based on mechanism of injury, anatomical structures involved, fracture complexity, and impact on orbital volume [11-13].

Classification type	Subcategories	Key features
Mechanism of injury	Blunt	Contusions, hematomas, fractures (e.g., blow-out fractures)
	Penetrating	Breach of skin/tissues, high risk of ocular damage (e.g., open-globe injuries)
Structures involved	Isolated soft tissue	Hematomas, edema, no bony involvement
	Orbital fractures	Floor, medial wall, lateral wall, roof; comminuted or linear
Complexity of fractures	Simple (e.g., linear)	Less severe, often managed conservatively
	Complex (e.g., comminuted)	May require surgical intervention
Impact on orbital volume	Blow-out	Increased orbital volume, risk of enophthalmos
	Blow-in	Decreased orbital volume, risk of exophthalmos

Preoperative assessment

Clinical and Imaging Assessment

Preoperative evaluation begins with a thorough clinical examination, including slit-lamp inspection of the anterior segment, though its effectiveness may be limited in severe trauma cases where visualization is obscured [22]. Computed tomography (CT) is the definitive imaging modality, essential for diagnosing orbital fractures, identifying intraorbital foreign bodies, and assessing soft tissue injuries [14,23]. High-resolution CT with multiplanar reconstructions provides detailed visualization of the bony orbit, anterior chamber, and posterior globe, serving as the gold standard for evaluating fracture complexity and guiding surgical decisions [23]. CT-derived parameters, such as fracture size and location, predict outcomes like enophthalmos, directly influencing the timing and nature of the reconstructive intervention [14,24]. Magnetic resonance imaging (MRI) complements CT by offering superior soft tissue resolution, particularly for optic nerve injuries or entrapment, though its use is limited when metallic foreign bodies are suspected [14]. Other modalities, such as cone beam CT (CBCT), may serve as alternatives for isolated orbital floor fractures but are less reliable in complex cases [25].

Multidisciplinary Evaluation

Given the periorbital region's intricate anatomy and functional demands, consideration for a multidisciplinary evaluation involving ophthalmology, oculoplastic surgery, plastic surgery, and/or maxillofacial surgery is advised, depending on the clinical scenario. This collaborative approach, when appropriate, leverages diverse expertise to address the multifaceted challenges of periocular trauma. For instance, in managing orbital lesions, teamwork enhances preoperative planning and surgical precision, minimizing complications and improving aesthetic results [26]. In naso-orbital-ethmoid fractures, digital-assisted multidisciplinary strategies have shown superior functional outcomes compared to single-specialty efforts [27].

Impact of Patient-Specific Factors on Reconstructive Planning

Patient-specific factors, including comorbidities, age, and gender, profoundly influence reconstructive planning and surgical success. Conditions like diabetes, smoking, and vascular diseases impair healing and increase complication risks, such as infections and flap necrosis [28,29]. While age alone does not significantly elevate complication rates, older patients often present with more comorbidities, amplifying perioperative risks [30,31]. Younger patients typically exhibit better functional recovery, such as improved eye motility after orbital fracture repair [32]. Gender differences may also affect complication risks, possibly due to variations in skin properties or vascularity [33].

Role of Imaging in Preoperative Planning and Intraoperative Guidance

Advanced imaging technologies, such as 3D CT and MRI, are integral to preoperative planning and intraoperative guidance. 3D CT enables precise fracture assessment and virtual surgical simulations, improving implant positioning accuracy [34]. Intraoperative CT provides real-time feedback, allowing immediate adjustments and reducing revision rates [35,36]. MRI-generated 3D models offer comparable accuracy to CT for orbital volume reconstruction, proving valuable when soft tissue detail is critical [37]. Intraoperative image guidance systems, integrating CT and MRI data, enhance precision with real-time navigation, particularly in complex resections and reconstructions [38,39]. Various imaging modalities play critical roles in the assessment and management of periocular trauma. Table 2 outlines their primary applications and benefits.

**Table 2:** Imaging modalities in periocular trauma assessment This table summarizes the imaging modalities employed in the assessment of periocular trauma, outlining their primary indications and key advantages for diagnosis, surgical planning, and intraoperative decision-making. CT, computed tomography; MRI, magnetic resonance imaging; 3D CT, three-dimensional computed tomography; CBCT, cone-beam computed tomography.

Imaging modality	Primary use	Key advantages
CT	Bony fractures, soft tissue injuries, foreign bodies	High-resolution, multiplanar reconstructions, guides surgical planning
MRI	Soft tissue evaluation, optic nerve injuries	Superior soft tissue contrast, useful for tumor infiltration
3D CT	Preoperative planning, virtual surgery	Enhances implant positioning accuracy, reduces revision rates
Intraoperative CT	Real-time assessment during surgery	Allows immediate adjustments, improves outcomes
CBCT	Isolated orbital floor fractures	Alternative to CT, though less reliable for complex cases

Timing and indications for reconstruction

Rationale for Immediate Versus Delayed Reconstruction

The choice between immediate and delayed reconstruction depends on the severity of the injury and its specific clinical features. Immediate reconstruction, typically within 48 hours to one week post-injury, is indicated for cases with extraocular muscle (EOM) entrapment or extensive orbital defects. Early intervention reduces the risk of complications like diplopia and enophthalmos by preventing fibrosis and scarring [40,41]. Delayed reconstruction, often up to four weeks post-injury, may be preferred for less severe fractures or when symptoms like diplopia persist, allowing for spontaneous recovery and avoiding unnecessary procedures [41,42]. For complex injuries, single-stage delayed reconstruction can achieve satisfactory outcomes with low complication rates [43]. Additional factors, such as logistical constraints or adjunctive therapies, may also influence timing without compromising results [44].

Conservative Management Versus Urgent Surgical Intervention

**Figure 4:**
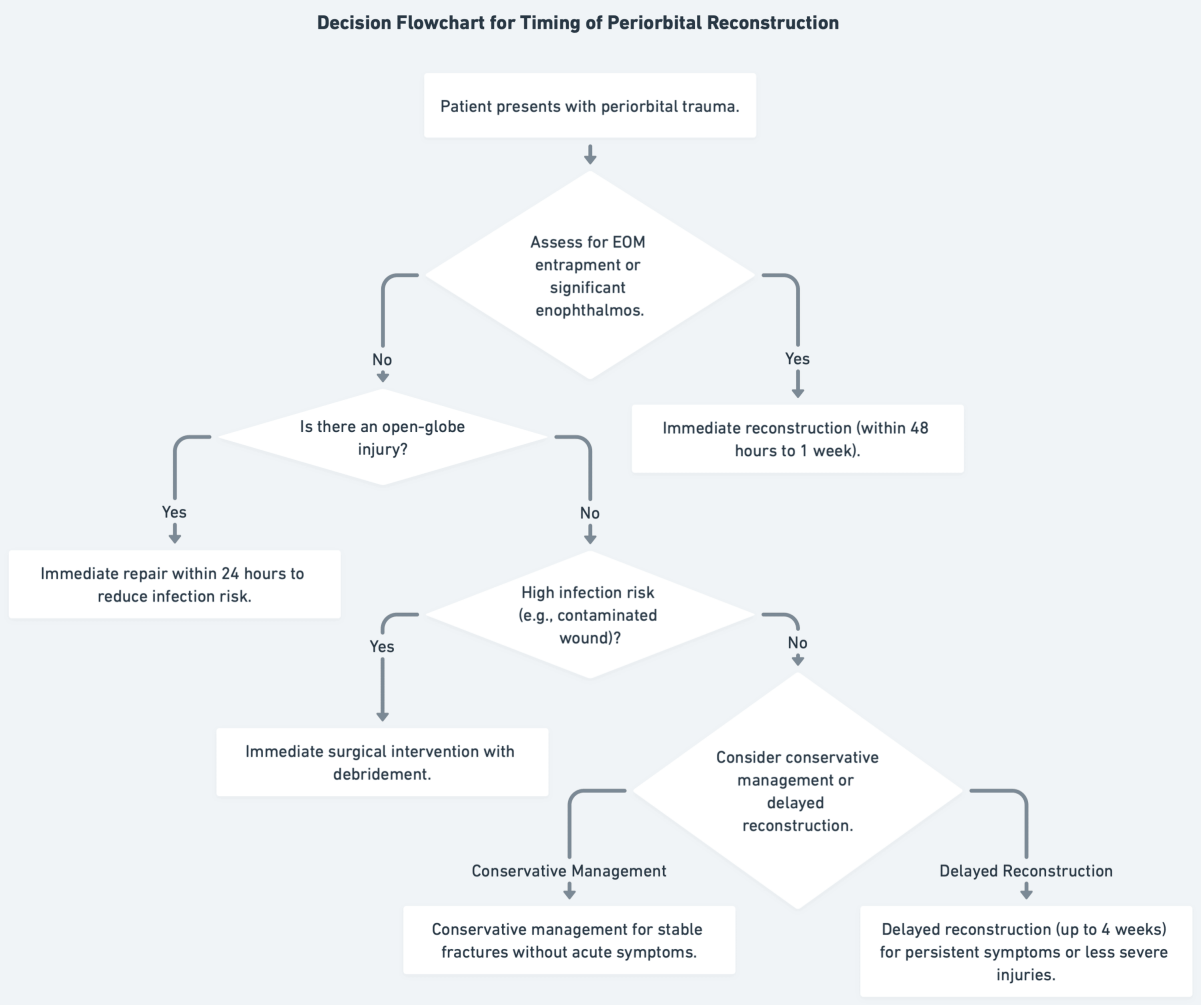
Decision flowchart for timing of periorbital reconstruction This figure illustrates a clinical flowchart guiding the timing of periorbital reconstruction based on key factors such as extraocular muscle (EOM) entrapment, significant enophthalmos, open-globe injury, and infection risk (e.g., contaminated wounds), recommending immediate intervention (within 24-48 hours to 1 week), conservative management for stable cases, or delayed reconstruction (up to 4 weeks) for persistent or less severe symptoms.

Conservative management, wound care and observation, is a viable option for uncomplicated orbital fractures without EOM entrapment or significant enophthalmos, as it may allow spontaneous resolution of motility disorders [42]. However, urgent surgery is essential when delay risks irreversible damage. For open-globe injuries, primary repair within 24 hours significantly reduces endophthalmitis risk [45]. Similarly, orbital fractures with EOM entrapment or severe diplopia benefit from surgery within two days to improve functional recovery [40,46]. The decision thus relies on evaluating clinical stability, acute complications, and the potential for natural recovery, with early intervention favored in high-risk cases (Figure 4).

Reconstructive techniques and approaches

**Figure 5:**
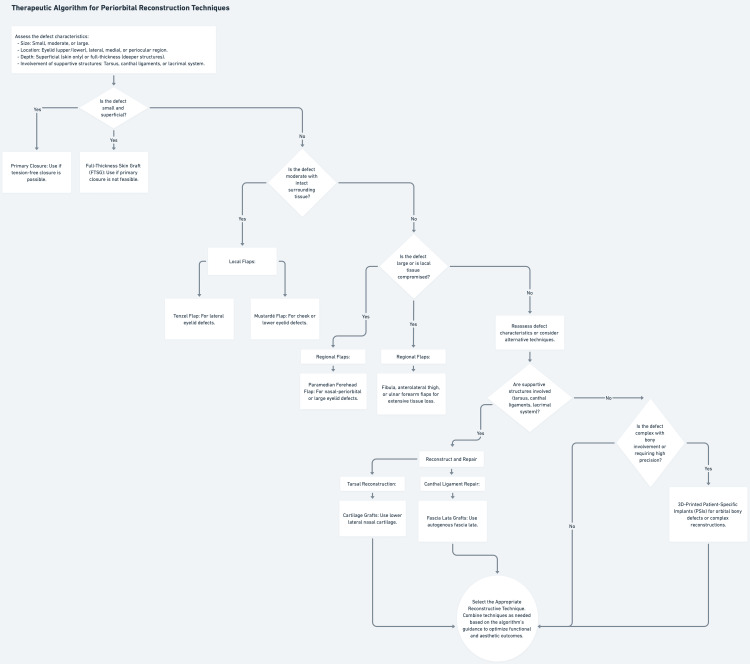
Algorithmic approach to periorbital reconstruction techniques This figure presents a therapeutic decision tree for selecting periorbital reconstruction methods, starting with defect assessment (e.g., size, superficiality, tissue loss) and branching to options like primary closure, skin grafts, local/regional flaps, microvascular transfers, supportive structure repairs (e.g., tarsal reconstruction, canthal ligament repair), or advanced technologies (e.g., 3D-printed implants), ensuring tailored functional and aesthetic outcomes.

Periorbital reconstruction after facial trauma requires surgical precision, anatomical expertise, and tailored techniques to restore function and aesthetics. This section explores key strategies, surgical principles, flaps, grafts, microvascular techniques, supportive structure management, and emerging technologies, assessed for their effectiveness based on the characteristics of the defect (Figure 5).

Fundamental Surgical Principles

Successful periorbital reconstruction depends on restoring lamellar integrity, maintaining eyelid position, and ensuring corneal protection. The eyelid’s anterior (skin and orbicularis oculi) and posterior (tarsus and conjunctiva) lamellae must be repaired, with at least one vascularized for viability [19,47]. Eyelid position is preserved by reattaching canthal tendons or using periosteal flaps to prevent malpositions like ectropion [1,48]. Corneal protection relies on techniques ensuring complete closure, such as grafts or flaps supporting blinking [47,49].

Local and Regional Flaps

**Figure 6:**
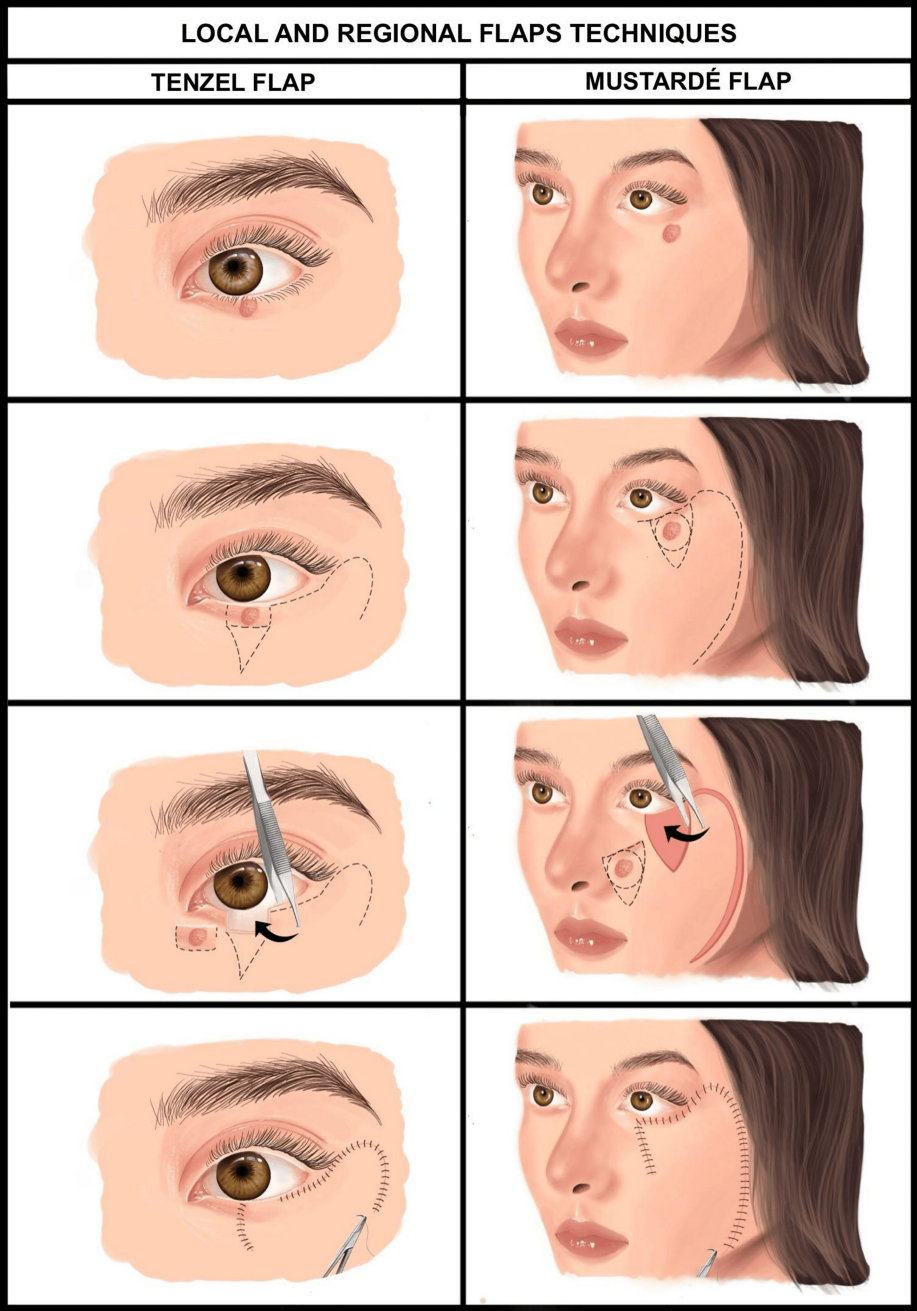
Local and regional flaps for periocular reconstruction This figure compares the Tenzel flap (left column) and Mustardé flap (right column) techniques. Panels depict the initial defect, surgical planning with incision markings, flap mobilization during surgery, and final sutured reconstruction, highlighting their application for eyelid and cheek defects to achieve functional and aesthetic restoration. Image credit: Created by Katherine López Soto using Procreate (Hobart, Australia).

Local and regional flaps adapt to defect size and tissue availability. Local flaps like the Tenzel flap, effective for lateral eyelid defects, and the Mustardé flap, ideal for larger cheek or lower eyelid repairs, offer an excellent tissue match [50]. The paramedian forehead flap suits extensive defects, providing durability and aesthetic integration [50]. Selection prioritizes vascularity and cosmetic outcomes [51] (Figure 6).

Skin Grafts Versus Locoregional Flaps

Skin grafts suit superficial defects, while locoregional flaps address deeper ones. Full-thickness skin grafts (FTSGs) excel in the periorbital area for their color match and low contraction, outperforming split-thickness grafts [52,53]. Flaps provide structural support and better aesthetics for complex defects [52]. FTSGs are preferred for eyelid resurfacing [54].

Free Flaps and Microvascular Techniques

Free flaps, like the fibula or anterolateral thigh, reconstruct large or complex defects with high success rates [55]. The lateral upper arm flap aids contracted eye sockets, offering pliable tissue [56]. These techniques, requiring microsurgery, deliver customized solutions for severe cases.

Management of Supportive Structures

Supportive structures, tarsus, canthal ligaments, and lacrimal system, require precise reconstruction. Lower lateral nasal cartilage substitutes tarsus effectively [57], while fascia lata grafts support the medial canthal ligament [58].

Newer Technologies: 3D-Printed Implants and Synthetic Scaffolds

3D-printed patient-specific implants (PSIs) offer precise fits for orbital defects, reducing complications [59,60]. Synthetic scaffolds enhance bone integration but face cost and delay issues [61,62].

Scar Formation and Management

Periorbital scarring, due to the thin skin, poses unique challenges. Subciliary incisions minimize visibility, while other approaches risk conspicuous scars [63]. Tension-free closure and laser therapy help preserve function and aesthetics.

Special Considerations in Periocular Reconstruction

Eyelid Margin Reconstruction for Function and Aesthetics

**Figure 7:**
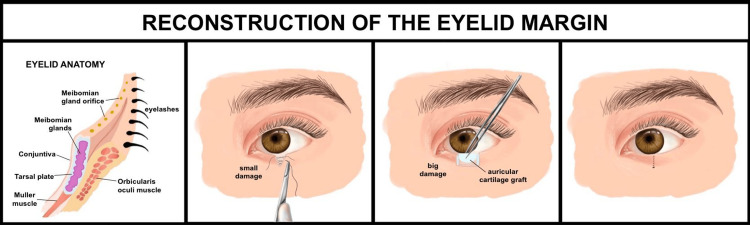
Reconstruction techniques for the eyelid margin defects This figure demonstrates the step-by-step reconstruction of the eyelid margin. The left panel shows normal eyelid anatomy in cross-section, including meibomian glands, conjunctiva, tarsal plate, and orbicularis oculi muscle. Subsequent panels illustrate repair of the small defects via direct suturing and the larger defects using an auricular cartilage graft for structural support, resulting in restored eyelid integrity. Image credit: Created by Katherine López Soto using Procreate (Hobart, Australia).

Reconstructing the eyelid margin must preserve both function and aesthetics, with approaches differing for upper and lower eyelids. For upper eyelid defects, small defects (<1/3) can often be closed directly, while larger defects (1/3 to 2/3) may require a myotarsocutaneous flap, and extensive defects (>2/3) often need a Cutler-Beard flap with auricular cartilage [64]. A single-stage advancement flap with cartilage grafts is also effective for full-thickness upper eyelid defects [65]. Lower eyelid reconstruction is more prone to ectropion; defects >1/3 typically require a double mucosal and myocutaneous island flap, while those >50% may use a Tripier flap with a conchal graft [64]. Techniques like the Hughes flap with a swing skin flap or periosteal flaps offer reliable outcomes for full-thickness lower eyelid defects [9,66]. For medial or central lower eyelid defects, the modified Mustardé flap with periosteal support provides a robust single-stage solution [48]. Technique selection must be tailored to defect size, location, and structural involvement (Figure 7). 

Recommended reconstructive techniques for eyelid margin defects vary by location and size, with key considerations for optimizing outcomes (Table 3).

**Table 3:** Reconstructive techniques for eyelid margin defects This table summarizes the recommended reconstructive techniques for upper and lower eyelid defects according to defect size and location, outlining key surgical considerations to optimize functional and aesthetic outcomes.

Defect location	Defect size	Recommended technique	Key considerations
Upper eyelid	<1/3	Direct closure	Leverages skin elasticity
Upper eyelid	1/3 to 2/3	Myotarsocutaneous flap	Restores both lamellae
Upper eyelid	>2/3	Cutler-Beard flap + Auricular cartilage	Comprehensive lamellar support
Lower eyelid	>1/3	Double mucosal + Myocutaneous island flap	Prevents ectropion
Lower eyelid	>50%	Tripier flap + Conchal graft	Balances aesthetics and stability
Lower eyelid (full-thickness)	Variable	Hughes flap + Swing skin flap	Reliable functional/aesthetic outcome

Restoration of the Lacrimal Drainage System

Preserving or restoring the lacrimal drainage system is essential to prevent chronic epiphora. Dacryocystorhinostomy (DCR) is the gold standard for nasolacrimal duct obstructions, with ~90% success rates for both external and endoscopic approaches [67]. Endonasal DCR is effective for dacryolithiasis [68]. In pediatric cases, probing resolves 83% of congenital obstructions, while silicone stenting may be used post-DCR in adults [69]. For proximal canalicular obstructions, trephination and intubation have failure rates of 17-22% [70]. Non-surgical options, like massage and antibiotics, can resolve up to 33% of pediatric epiphora cases [69]. Balloon dacryoplasty is less effective than DCR [67]. The selection of the method should consider obstruction site, etiology, and patient factors, with postoperative care crucial to prevent recurrence.

Surgical Modifications and Adjuncts in Older or Severely Traumatized Patients

In older or severely traumatized patients, adjunctive procedures like brow suspension and canthopexy address tissue laxity or structural deficits. Brow suspension, using brow fat pad sutures, enhances outcomes in patients with brow ptosis [71]. Canthopexy supports mild-to-moderate lower eyelid laxity, while canthoplasty (e.g., lateral tarsal strip) is reserved for severe cases [72]. Canthopexy can also restore function in traumatic upper eyelid ectropion [73]. Medial canthopexy, though less common, may be indicated but has higher revision rates in older patients [74]. These adjuncts balance functional and aesthetic needs, especially in compromised tissues.

Perioperative and Postoperative Management

Infection Prophylaxis and Inflammation Control

Preventing infection and controlling inflammation are essential for successful periorbital reconstruction. Intraoperative protocols involve meticulous techniques, including hair removal with clippers, chlorhexidine-alcohol skin preparation, and antibiotic prophylaxis within 60 minutes of incision, alongside normothermia and glucose control [75,76]. Postoperatively, maintaining glucose levels (140-180 mg/dL) and normothermia, with negative pressure wound therapy for complex cases, further mitigates the risk of surgical site infection (SSI) [77]. Perioperative corticosteroids reduce early edema and ecchymosis, enhancing recovery [78].

Perioperative Treatments

Strategic treatments improve outcomes. Corticosteroids attenuate inflammation and may reduce SSIs, though their role in complex reconstructions is debated [79]. Intracameral antibiotics outperform topical agents in preventing endophthalmitis, suggesting benefits for periorbital cases [80]. Ocular lubricants maintain corneal health, preventing dryness and epithelial damage [80].

Postoperative Care

Postoperative care emphasizes wound management and protection. Wounds are kept clean and dry, with sutures removed within five to seven days to minimize scarring. Protective eye shields prevent trauma during early healing, crucial due to the region’s exposure [81].

Management of Complications

Common complications, graft/flap necrosis, infection, eyelid malposition, and corneal exposure, require prompt management. Necrosis necessitates vascular assessment and potential debridement [82,83]. Infections are treated with broad-spectrum antibiotics, adjusted per culture, with debridement for severe cases. Eyelid malposition, like ectropion, may require canthoplasty [82,84]. Corneal exposure demands aggressive lubrication or tarsorrhaphy [82,85].

Multidisciplinary collaboration

Scenarios Requiring Multidisciplinary Collaboration

Effective periocular injury management relies on coordinated efforts from oculoplastic surgeons, maxillofacial surgeons, and ear, nose, and throat (ENT) specialists. Oculoplastic surgeons address eyelid, lacrimal, and orbital injuries, crucial in cases like high-pressure injection trauma to preserve vision. Maxillofacial surgeons reconstruct midface and orbital fractures, using tools like virtual surgical planning for structural and aesthetic restoration, especially in complex cases like orbital roof fractures [86,87]. ENT specialists manage sinonasal involvement or airway compromise, supporting eye-sparing interventions [88]. This unified approach mitigates risks of vision loss, deformity, and airway obstruction [87,88].

Benefits of Interdisciplinary Team Approaches

Interdisciplinary collaboration enhances patient care through meticulous preoperative planning and synchronized surgery, reducing complications. Technologies like image-guided navigation ensure precise anatomical restoration [89]. Single-stage reconstruction of bony and soft tissue defects minimizes complications and accelerates recovery [43]. Combined follow-up protocols enable early complication detection, optimizing long-term outcomes [87].

Role of Non-Surgical Specialists in Rehabilitation

Non-surgical specialists, physical therapists, occupational therapists, and psychologists, are vital for holistic recovery. Physical therapists facilitate facial muscle reeducation, enhancing function in severe cases [90]. Techniques like hydrocortisone iontophoresis improve cranial nerve injury symptoms [91]. Occupational therapists adapt activities to limitations, while psychologists address the emotional impact, ensuring comprehensive recovery.

Perspectives

Current Best Practices and Their Evolution

Modern periorbital reconstruction employs defect-specific strategies for optimal outcomes. Eyelid reconstruction follows an algorithmic approach: small defects (<1/3 of the eyelid) allow primary closure, while larger defects require mucosal-myocutaneous flaps or total lid reconstruction using Fricke flaps and tarsoconjunctival grafts [64]. Extensive defects benefit from microvascular free tissue transfer, such as fibula or anterolateral thigh flaps [55]. Orbital wall reconstruction leverages biocompatible materials, though material selection lacks consensus [92]. Intraoperative image guidance, notably CT, enhances implant accuracy and reduces revisions [38]. These trends highlight patient-specific, technology-driven approaches.

Knowledge Gaps and Future Research Directions

Key gaps remain unresolved. Standardized outcome measures for aesthetic and functional results are lacking, hindering technique comparisons [53]. Optimal timing for non-urgent repairs needs evidence-based guidelines [42]. The soft tissue management’s impact on outcomes is underexplored [93]. Regenerative technologies, like 3D bioprinting, require clinical validation [94]. Flap technique comparisons (e.g., temporal vs. forehead flaps) are needed to assess complications [95], and revision surgery predictors remain understudied [96]. Multicenter prospective studies are critical.

## Conclusions

Periocular reconstruction following facial trauma is a complex field that demands a multidisciplinary approach and a deep understanding of the periocular region’s anatomy and function. However, challenges remain, including the lack of standardized outcome measures and the need for further research on optimal reconstruction timing. Collaboration among plastic surgeons, ophthalmologists, maxillofacial surgeons, and other specialists is vital to optimizing patient outcomes. Addressing knowledge gaps through prospective, multicenter studies will refine clinical practices and enhance patient care. Continued innovation and research are essential to overcome these challenges and ensure that reconstructive efforts not only restore form but also preserve critical ocular functions.
